# Tetralogy of Fallot With Absent Left Pulmonary Artery in a Patient Who Passed Critical Congenital Heart Disease Screening

**DOI:** 10.7759/cureus.30604

**Published:** 2022-10-23

**Authors:** Ruth Y Eletta, Isra Idris, Abinash Pandey, Swetha Gannarapu, Endurance O Evbayekha

**Affiliations:** 1 Pediatrics, NYC Health + Hospitals/Woodhull, New York CIty, USA; 2 Pediatrics, NYC Health + Hospitals/Woodhull, New York City, USA; 3 Internal Medicine, St. Luke's Hospital, St. Louis, USA

**Keywords:** tetralogy of fallot, critical congenital heart disease, neonatal screening, pediatric congenital heart disease, absent left pulmonary artery

## Abstract

The literature on tetralogy of Fallot (TOF) is abundant. It is a fairly common cyanotic congenital heart disease. It results from the anterior malalignment of the conal septum resulting in the aberrant formation of the ventricular septum leading to a defect. This presents a very important clinical significance because the prognosis usually depends on the clinical evaluation and initiation of timely therapy.

We present a two-week-old baby with normal birth history and an uncomplicated newborn nursery course. He also passed the Critical Congenital Heart Disease screen at 24 hours of life. The routine examination by the pediatrician led to further investigations and treatments, highlighting the importance of good history-taking and clinical examination skills.

## Introduction

Tetralogy of Fallot (TOF) is a prevalent form of cyanotic congenital heart disease. It is due to the anterior malalignment of the conal septum leading to a ventricular septal defect (VSD), and as it moves anteriorly, it pulls the aorta to override the septum. It also leads to a right ventricular outflow obstruction due to narrowing of the right ventricular outflow tract (RVOT) and right ventricular hypertrophy because the right ventricle contracts against a fixed obstruction [1]. The burden of TOF is significant for about 7-10% of all congenital heart diseases (CHDs). It is estimated to be the most common cyanotic congenital heart disease, requiring medical and surgical management, usually in the first year of life. Current advances in prenatal screening and fetal echocardiography have led to increased rates of prenatal diagnosis of TOF [2].

## Case presentation

We present a male newborn delivered to a 30-year-old lady via spontaneous vaginal delivery at 40 weeks following an unremarkable pregnancy course. An anatomy scan done in late pregnancy could not visualize the fetal heart; hence a fetal echo was recommended but not performed. The baby was born with a birth weight of 6lb8oz. He had poor feeding in the first few hours after birth, and a septic workup was done, which was negative.

On day one of life, Appearance, Pulse, Grimace, Activity, and Respiration (APGAR) scores were 9 in the first minute and 9 in 5th minute of life. He had poor feeding in the first few hours of birth and was tachypneic with an episode of desaturation to 89-90 % on room air, he received continuous positive airway pressure (CPAP) and high flow nasal cannula. Tachypnea resolved after 12 hours of life, with no repeat desaturation episode. Cardiovascular examinations showed normal heart sounds of s1 and s2, with no murmur. Other physical examination findings were within normal limits. 

At 24 hours of life, a critical congenital heart screening was done, with the pulse oximeter placed on the right hand (pre-ductal) and left foot (post-ductal) he had a saturation of 97% both pre and post-ductal. He was discharged home in stable condition. He was doing well and feeding well, with no history of tachypnea, fatigue, cyanosis, or diaphoresis.

On day 10 of life, he was taken for a routine visit at the primary care doctor's office, a grade 3/6 systolic murmur was heard, and he was referred to the cardiologist for further evaluation. He was seen by the cardiologist on the 14th day of his life. The cardiologist reported a harsh 3/6 systolic murmur at the left upper sternal border. Capillary refill was normal, less than wo seconds. The radial and femoral pulses were normal, with no radio-femoral delay. Vital signs at the cardiologist visit were; pulse rate (PR)- 142 beats per minute (bpm), temperature- 97.7 Fahrenheit, SPO2- 98% in room air, respiratory rate (RR)- 40 cycles per minute (cpm), all within the normal limit of expected for age.

**Figure 1:**
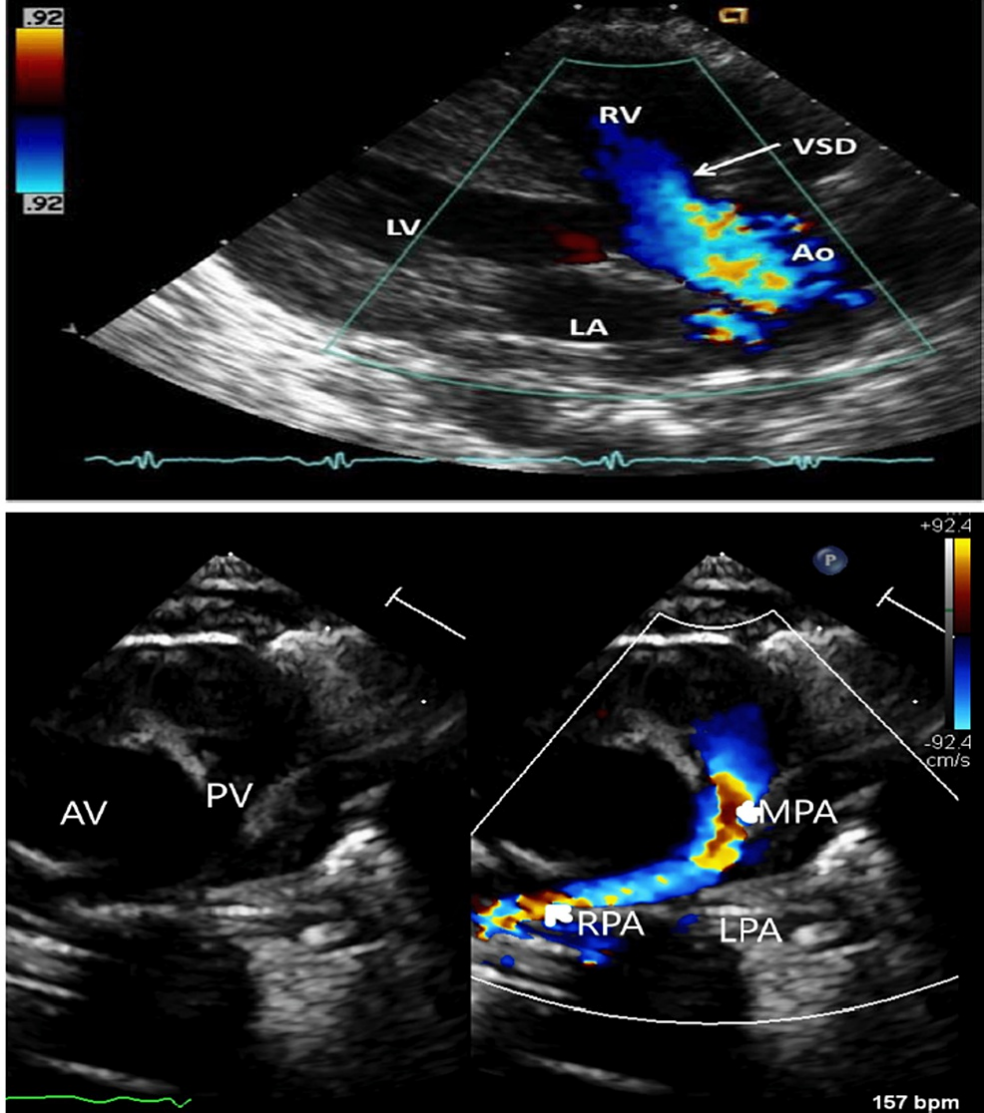
Echocardiogram Showing Blood Flow AV= Aortic Valve; PV= Pulmonic Valve; VSD= Ventricular Septal Defect; LV= Left Ventricle; RV= Right Ventricle; RA= Right Atrium; Ao= Aorta; RPA= Right Pulmonary Artery; LPA= Left Pulmonary Artery; MPA= Middle Pulmonary Artery

The pediatric cardiologist performed echocardiography which demonstrated the TOF with right ventricular outflow tract (RVOT) obstruction, mildly hypoplastic main and right pulmonary artery, and diminished left-sided venous return compared to the right as seen in Figure 1. The transthoracic echocardiography did not visualize the left pulmonary artery (LPA). 

The child was referred to the emergency department where a heart murmur was noted. It was also noticed that he had perioral cyanosis while crying. He was transferred to the critical care cardiovascular intensive care unit (CCVICU) for further evaluation and management.

He underwent diagnostic catheterization, which confirmed the diagnosis. He was commenced on a high dose of prostaglandin in an attempt to reopen the patent ductus arteriosus (PDA). No improvement was seen in echocardiography's left pulmonary artery caliber despite the high dose of prostaglandin.

**Figure 2:**
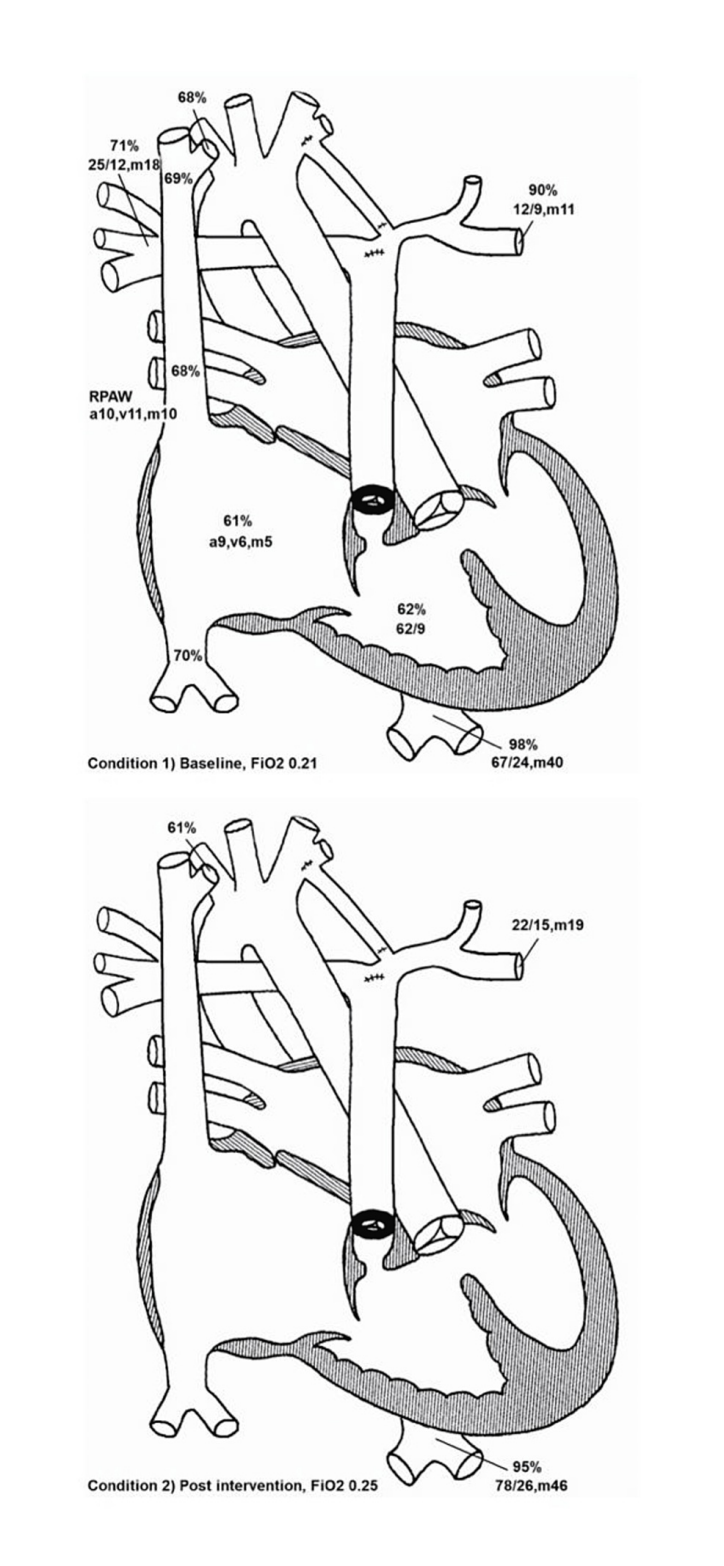
Pre- and Post-Cardiac Catheterization Procedure Intra-Operative Findings: Hypertrophied right ventricle with normal qualitative systolic function, Dynamic subvalvar pulmonary stenosis, Hypoplastic pulmonary valve annulus with a caliber of approximately 5.9mm, Hypoplastic main pulmonary artery with a caliber of 4.8mm, Confluent branch pulmonary arteries Proximal right pulmonary artery caliber of 8.7mm, Proximal left pulmonary artery caliber of 2.4mm, with distal reference vessel caliber of 5.7mm. There was significant interval growth of distal artery vasculature

On day 17, cardiac catheterization confirmed the previous diagnosis made by an echocardiogram, as seen in Figure 2. There appeared to be a trivial collateral vessel between the left pulmonary vein system and a tiny LPA. Prostaglandin was re-initiated at a very high dose to re-open the ductus arteriosus (DA) that appeared to supply the LPA. On the day of life 23, a left modified Blalock-Taussig-Thomas (BTT) shunt was inserted, and the ductus arteriosus was ligated, with direct unifocalization of LPA. 

Why Blalock-Taussig-Thomas (BTT) shunt?

It is usually more challenging to put in a patent ductus arteriosus (PDA) stent because, with it, one cannot limit pulmonary blood flow. Also, because it's temporary and one has to go in later to take out the stent. Sometimes, the follow-up surgery is challenging because some of the stents might have adhered to the wall of the vessels. Hence, we opted for the BTT because our patient was relatively stable.

## Discussion

TOF combines four key features: VSD, pulmonary stenosis, right ventricular hypertrophy, and a dextro-positional aorta causing an override of the ventricular septum by the aortic root. In addition to being an anatomic anomaly, TOF also seems to be a complicated genetic disorder associated with other anomalies such as absent pulmonary valves, absent/hidden pulmonary arteries, and extracardiac anomalies [3].

Obstruction to the pulmonary artery blood flow is usually at the subpulmonic area and the pulmonary valve. The main pulmonary artery might be small, and various degrees of branch pulmonary artery stenosis may be present. The degree of right ventricular outflow tract (RVOT) obstruction determines the age of presentation, with the most extreme being TOF with pulmonary atresia or critical RVOT obstruction (these would be ductal dependent) to very mild or no RVOT obstruction ("pink" tet) [2,3]. 

In TOF, the anatomy of branch pulmonary arteries can be variable, from continuous pulmonary arteries to ductal-dependent branch pulmonary arteries to major aortopulmonary collateral arteries (MAPCAS). Occasionally, as seen in our case, the branch pulmonary artery may not be visible. On such occasions, pulmonary blood flow may be supplied by a patent ductus arteriosus (PDA) or multiple aortopulmonary collateral arteries that arise from ascending or descending aortas.

Unilateral absence of the left pulmonary artery (LPA) is five to eight times more frequent than the right pulmonary artery [3]. Some patients with the impression of an absent pulmonary artery might have a concealed pulmonary artery (normal size or small) with a closed communication between the ductus arteriosus and pulmonary artery [2,3]. The initial treatment of TOF varies depending on the RVOT obstruction. Treatment of ductal-dependent TOF is to avoid rapid deterioration as the ductus begins to close. This is provided by the intravenous administration of prostaglandin E1 (PGE1), which relaxes smooth muscles, dilates ductus arteriosus, and provides pulmonary blood flow. Surgical treatment is either palliative (Blalock-Taussig-Thomas shunt) or corrective surgical therapy [3,4].

In corrective surgery, VSD is corrected with patch closure. RVOT obstruction is relieved by resecting obstructive muscle bundles if any, and patching of the RVOT. Occasionally, pulmonary valvotomy and a trans-annular patch are required. In cases where one of the pulmonary arteries is absent, ductus arteriosus stenting by passing a guidewire toward the concealed pulmonary artery might help rehabilitate the pulmonary artery's small/diminutive branch [5].

## Conclusions

Our two-week-old baby had a normal birth and an uncomplicated newborn nursery course. He also passed the critical congenital heart disease screen at 24 hours of life. While growing and developing normally, he presented with a murmur, which prompted further evaluation. This report emphasizes that despite passing the critical congenital heart disease screening, physicians should be thorough in evaluating the newborn as a good history and physical examination are key to early diagnosis when combined appropriately with imaging.
